# Soft-Tissue Material Properties and Mechanogenetics during Cardiovascular Development

**DOI:** 10.3390/jcdd9020064

**Published:** 2022-02-21

**Authors:** Hummaira Banu Siddiqui, Sedat Dogru, Seyedeh Samaneh Lashkarinia, Kerem Pekkan

**Affiliations:** 1 Department of Mechanical Engineering, Koc University, Istanbul 34450, Turkey; hsiddiqui20@ku.edu.tr (H.B.S.); sdtdogru@gmail.com (S.D.); lashkarinia.samane@gmail.com (S.S.L.); 2 Department of Biomedical Engineering, Boston University, Boston, MA 02215, USA; 3 Department of Bioengineering, Imperial College London, London SW7 2BX, UK

**Keywords:** cardiovascular microstructure, congenital heart defects, soft-tissue mechanics, strain energy, residual stresses, cardiovascular development, chick embryo, cardiovascular system, hemodynamics, arterial pressure, cardiac output, embryonic development, optical coherence tomography, heart-valve development, embryonic heart

## Abstract

During embryonic development, changes in the cardiovascular microstructure and material properties are essential for an integrated biomechanical understanding. This knowledge also enables realistic predictive computational tools, specifically targeting the formation of congenital heart defects. Material characterization of cardiovascular embryonic tissue at consequent embryonic stages is critical to understand growth, remodeling, and hemodynamic functions. Two biomechanical loading modes, which are wall shear stress and blood pressure, are associated with distinct molecular pathways and govern vascular morphology through microstructural remodeling. Dynamic embryonic tissues have complex signaling networks integrated with mechanical factors such as stress, strain, and stiffness. While the multiscale interplay between the mechanical loading modes and microstructural changes has been studied in animal models, mechanical characterization of early embryonic cardiovascular tissue is challenging due to the miniature sample sizes and active/passive vascular components. Accordingly, this comparative review focuses on the embryonic material characterization of developing cardiovascular systems and attempts to classify it for different species and embryonic timepoints. Key cardiovascular components including the great vessels, ventricles, heart valves, and the umbilical cord arteries are covered. A state-of-the-art review of experimental techniques for embryonic material characterization is provided along with the two novel methods developed to measure the residual and von Mises stress distributions in avian embryonic vessels noninvasively, for the first time in the literature. As attempted in this review, the compilation of embryonic mechanical properties will also contribute to our understanding of the mature cardiovascular system and possibly lead to new microstructural and genetic interventions to correct abnormal development.

## 1. Introduction

Embryonic cardiovascular development is an intriguing and vital process. This paper presents a comparative review of the material properties in the embryonic and fetal cardiovascular systems of different model organisms at consequent stages. An integrated and time-lapsed biomechanical and biochemical perspective of embryonic vascular remodeling and vascular growth is central to this review effort. In addition, the signaling pathways that ultimately regulate mechanical properties of major components of the dynamic vascular phenotype are also revisited. Recent advances in embryonic animal models of altered mechanical environment and increasing access to the mRNA interventions also motivated this review. It is expected that targeted combination of both interventions will start to emerge in the literature. Furthermore, the material properties such as stiffness [1], stress–strain measurements [1], strain rate [2], and elasticity [3] enunciate the structural and functional properties of the cardiovascular system and can help identify the emergence of congenital heart defects (CHDs). Shear stress-responsive elements stimulated by blood flow cause cellular activities; hence, the study of resulting mechanical properties would also provide insight into the functional evolution of the developing system [4]. Critical functions such as apoptosis, organogenesis, epithelial-mesenchymal transition during heart-valve development, and associated gene expressions are influenced or directly caused by the mechanical stresses [5]. Elasticity plays a crucial role in the efficient pumping mechanics of the ventricles [6]. Mechanical factors such as myocardial wall stress and strain [7,8], hemodynamics, and ventricular pressure [9,10,11] are also known to interfere with cardiac growth and development.

The stages of development of the embryo play a vital role in the targeted mechanical properties and their rate of change. System-level cardiovascular properties such as stiffness, compliance, and vascular resistance are significantly altered as the embryo develops [12]. While several empirical correlations have been drawn between the mechanical and morpho-structural properties of cardiovascular components [13], the properties are nonlinear and, hence, require a foundation of soft-tissue mechanics and custom experimental tools [3]. The survival and sustainability during embryogenesis may require a tight balance between the hemodynamic parameters and material characteristics [4]. Despite the overall changes during long-term development, some parameters such as stress–strain characteristics, stiffness, and stretch values tend to remain constant with growth, as demonstrated in key cardiovascular components [1]. Mechanical characterization is critical to detect cardiac anomalies with clinical significance at the early stages. An abnormality such as increased or decreased stresses, strains, or other mechanical factors can cause a fluctuation in the upstream transcription factors, which are indicative of the prevalence of cardiac defects [14,15,16,17,18,19]. Well-established transcription factors, such as KLF-2, ET1, and NOS3, are activated by wall shear stress [20,21,22]. Abnormalities in mechanical properties of the looping ventricle also have the potential to trigger CHDs [8,23].

The biomechanics of cardiovascular development was initially investigated by a few pioneering research groups. Leading publications of these teams guided the present review systematics by providing the seed papers. Due to the authors’ expertise and research interests, the recent experimental manuscripts published in specialized journals were screened from a biomedical and biomechanical engineering perspective. In this comparative review, for each species, we classified the cardiovascular system on the basis of its major components and compiled the embryonic stage-specific mechanical properties as they significantly change during development. The Carnegie stages (CS), a standardized 23-stage scale (up to 8 weeks of the human gestation period), are used as a scale to compare the embryonic development between different organisms [24].

The present review of embryonic mechanical characterization indicated gaps in literature where further experiments are needed. Therefore, two novel and noninvasive mechanical characterization techniques are introduced here for the first time in the literature. These techniques are applied to early avian embryonic stages and have the potential to address some of the experimental challenges discussed in this article.

The review starts with the basic methodology for measuring the material properties. The properties for avian embryos at key developmental stages are presented in detail due to the availability of ample data for this established cardiovascular animal model. Corresponding properties are compiled for porcine, human, and rat cardiovascular systems, in sequence. Despite the limited data and the complex/transitionary circulation system, *Xenopus* species are included for completeness [25]. The genetic impact of mechanical properties is reviewed, and the known interactions between the governing genes and morphology are presented. Thus, this review aims to establish insight into the relationship among mechanical properties, morphological structure, and genetic characteristics of the cardiovascular system as it develops into its more complex and fascinating mature configuration across species.

## 2. Materials and Methods

### 2.1. Traditional Methods

Mechanical characterization includes basic mechanical testing and microscopic visualization, as summarized in Table 1. Experimental approaches include micropipette aspiration, optical stretching, optical tweezers, optical coherence tomography (OCT), micro cantilever-based techniques, pulsatile testing, and micro-indentation. These methods are used to observe mechanical factors such as stress-strain, elasticity, and strain energy function of soft tissues. The most popular and practical form of these techniques is tensile testing [13]. Tensile tests can be performed in a uniaxial or biaxial experimental setup to quantify the isotropic or anisotropic material properties of soft tissues, respectively [26,27,28,29]. However, they require the tissue to be extracted, and the quantification of small embryonic tissue properties locally via tensile testing is highly challenging, thus creating a niche for novel noninvasive methods.

To support the mechanical testing data, simultaneous visual observation of three-dimensional changes of the tissue microstructure is important. Quantitative visualization techniques include fluorescence microscopy, two-photon/confocal imaging, magnetic resonance imaging and echocardiography [34]. Some researchers, as in [47], incorporate optical microstructural tracking with mechanical testing such as uniaxial, biaxial, and multiaxial testing, for better inference. The samples are subjected to loading until complete rupture and the stress-strain curve/stiffness is acquired [13]. Developmental changes in material properties are best observed using advanced microscopy [42]. These techniques are often optimized for a specific tissue or target cardiovascular component [14]. Multiple methods are also employed simultaneously for cross-validation, as in [3]. For high-quality research data, in utero and in ovo approaches are strongly preferred over the ex vivo or in vitro cultures of the developing cardiovascular components.

Due to the exponential nature of vascular stress-strain curves, the hyper-elastic exponential material model [48] is adopted to characterize the embryonic tissue [49,50]. For example, the Ogden material model [51] is commonly used, as employed by Von Dassow et al. [52] and Yao et al. [8] to model *Xenopus laevis* embryonic tissue and looping heart tissue in chick embryo, respectively. A simplified form of the Ogden material model is the neo-Hookean strain energy function, which was used in our recent work to characterize the growth of aortic arch tissue at early embryonic stages of the chick embryo [45].

Another basic material model for mechanical characterization of the soft tissue is Fung’s material model [53,54]. Fung-type material is an anisotropic hyper-elastic constitutive model with the strain energy function described in [12]. Fung’s equation represents a pseudo-elastic stress-strain relation (Equation (1)) and, thus, can be used to identify the corresponding nonlinear material parameters if the stresses and strains are experimentally evaluated.

(1) $$\rho_{o}W=\frac{c}{2}expQ,$$

where $\rho_{o}$ is the mass density, *W* is the pseudo-strain energy per unit mass, *c* is a constant with a unit of kPa (stress), and *Q* is given by the following equation:

(2) $$Q=b_{1}E_{\theta }^{2}+b_{2}E_{z}^{2}+b_{3}E_{r}^{2}+2b_{4}E_{\theta }E_{z}+2b_{5}E_{z}E_{r}+2b_{6}E_{r}E_{\theta },$$

where *E_o_*, *E_z_*, and *E_r_* are Green’s strain components in the circumferential, longitudinal, and radial directions, respectively, while *b*_1_, *b*_2_, *b*_3_, *b*_4_, *b*_5_, and *b*_6_ are the nonlinear material parameters and are constants for a given material. The values of *b_n_* correspond to the tissue structure, which determines the behavior of the material, although an explicit physical mechanical parameter cannot be assigned to the constants. Most importantly, these material models can be implemented in finite element modeling (FEM) frameworks to solve the detailed stress/strain distributions in subject-specific computational models of developing cardiovascular components [55,56,57,58].

### 2.2. Novel Noninvasive Methods

The first noninvasive technique introduced in this manuscript simultaneously measures lumen diameter and pressure waveforms over the cardiac cycle using OCT (Thorlabs, Newton, NJ, USA) and a servo-null pressure system (WPI, Sarasota, FL, USA) respectively. These measurements are sufficient to estimate both the unloaded and the loaded Fung’s material parameters. Furthermore, the residual stresses can be acquired noninvasively as demonstrated in our earlier work applied to mature arteries [12]. For developing tissues, residual stresses are critical as their hyper-restoration is presumed to drive vascular growth [59]. In the present paper, we applied this technique to characterize embryonic pharyngeal arches and the vitelline arteries of early avian embryo using fertilized *Gallus gallus domesticus* eggs for Hamburger-Hamilton (HH) stages [60] HH16 to HH24. This noninvasive data are presented for the first time in the literature. The experiments were conducted on five embryos to obtain the vitelline artery parameters and on eight embryos to obtain the IVth aortic arch vessel parameters, including their residual stress components and distribution. Since these experiments were conducted in ovo, results include external mechanical effects from albumin, including surface tension, as the embryo is oriented close to the air sac.

**Figure 1:**
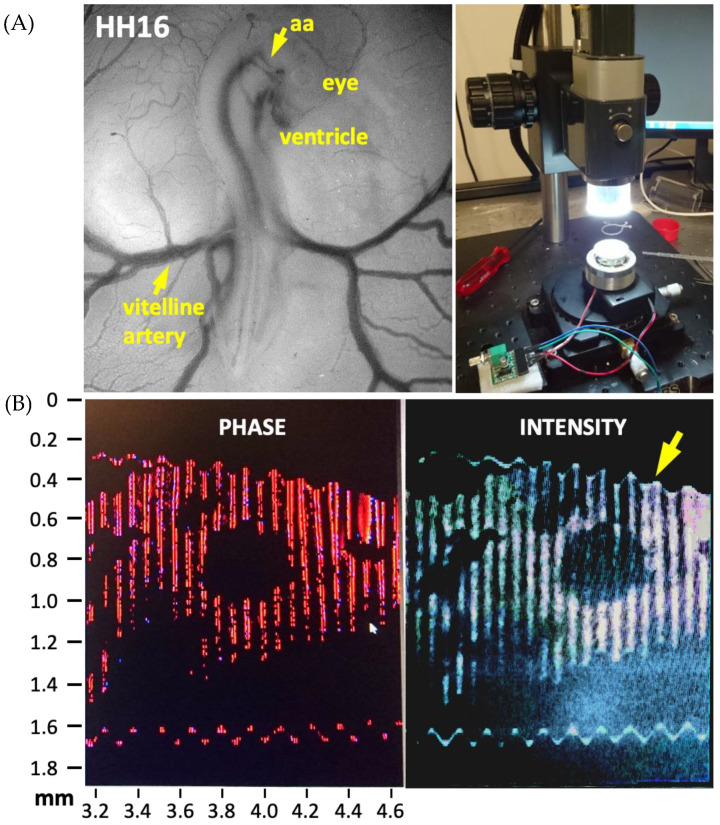
The experimental setup used to evaluate material properties in chick embryos at HH16 (vitelline artery) to HH24 (aortic arch, aa) is shown on the right. (**A**). Stage HH16 chick embryo imaged under a stereomicroscope is also provided. (**B**). Preliminary optical coherence tomography images of chick ventricle during acoustic forcing (0–20 kHz) performed for noninvasive elastography. A cross-section of the ventricle is displayed. The arrow points to the instantaneous deformation of the soft tissue.

Alternatively, as the next noninvasive approach, an actuator was used to create a wave of frequency in the range 0–20 kHz, as in tissue elastography [61]. A signal with a desired amplitude was generated and directed at the chick embryo in ovo. A change in direction of the incident wave was observed in the OCT Doppler mode, depending on the local material properties the wave is reflected by. The change in the direction of frequency was recorded in the form of an image. Optimization of frequency and amplitude of the generated wave provided image data amiable for further analysis. The darker and lighter sections in the image are representative of the material characteristics of the tissue. A preliminary experimental setup of this technique is provided in Figure 1.

## 3. Results

### 3.1. Avian Embryonic Development

Avian embryos, such as quail and chick, are the models of choice as they closely relate to the biventricular human cardiovascular system [62] and enable a large sample size. In the literature, material properties have been studied for a broad range of embryonic HH stages and cardiovascular components, as summarized below.

#### 3.1.1. Ventricles

Anatomical dimensions are first reviewed in order to provide an interpretation for the reported stress/strain levels and other mechanical properties. The left ventricle (LV) compact layer thickness, which excludes the trabeculations, is larger than that of the right ventricle (RV) from HH24 to HH34 [63]. Compact layer thickness is 45 μm and 35 μm at HH34 and HH24, respectively, for LV and 35 μm and 15 μm at HH34 and HH24, respectively, for RV. The total volume of the myocardium for a healthy embryo was observed to be ~0.7 mm^3^ and ~0.35 mm^3^ for the LV and RV, respectively, for combined compact and trabecular regions [63]. The LV wall thickness, including the trabeculae, is ~300 μm at HH27, and it progressively increases to ~440 μm at HH31 [63]. At HH29, the length of the RV and LV is approximately 1.25 mm and 1.75 mm, which increases to 3.5 mm and 5.5 mm at HH40, respectively [63]. Embryonic ventricular dimensions can also be estimated from published developmental atlases [64].

Earlier studies acquired the progressive changes in ventricular tissue strain values. For Leghorn chick embryo at HH16, micropipette aspiration was employed to measure the circumferential and peak axial Lagrange strain of the embryonic ventricle, indicating that the strains are different for systole and diastole, with the peak strain being −0.16 ± 0.08. There is a shortening of about 20% during systole [65]. Strain is higher near the boundaries of the primitive RV and reduces close to the mid-ventricle. Higher strain values imply potential for remodeling and enhanced biomolecular activity. The stiff myocardium supports the end-systolic pressure load indicated by the transmural stress distribution, accounting for the effects of residual strain. Residual stress levels significantly affect the stress distribution of the loaded state. For example, residual stress increases the stress concentration of the myocardial layer at HH17 [66]. For time-lapsed data, the noninvasive residual stress estimation approach introduced in Section 2.2 can also be applied to the ventricles.

**Figure 2:**
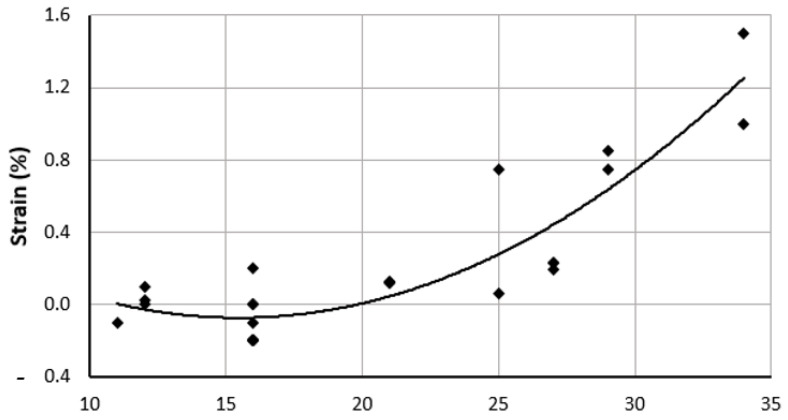
Overall strain trend of embryonic chick ventricles (LV and RV) presented as a percentage, as compiled from multiple literature sources, from HH11 to HH34. Corresponding references are cited in the text and in Table 2. HH: Hamburger-Hamilton stages.

At HH18, using uniaxial loading and FEM, the changes in the strain values between HH16 and HH18 were reported by Miller et al. [67]. The longitudinal Lagrangian strain of the ventricles was calculated from three microspheres at the top center of the outer curvature and reached a maximum value of 0.19 at HH18. The cross-sectional area at HH16 was observed to be 0.049 mm^2^ and that at HH18 was observed to be 0.059 mm^2^ [67], indicating an increase in the thickness of the ventricles. An increase in strain indicates an improvement in the ventricles’ capacity to withstand increased blood pressure during development. As the stages progress, the strain levels increase, as presented in Figure 2.

Cyclic uniaxial loading of the LV shown by Miller et al. [67] at HH16 showed that the longitudinal strain ranges from 0.0 to 0.25, circumferential strain ranges from −1.5 to −0.01, and shear strain remains approximately zero. Thus, circumferential strains are negative, and longitudinal strains are positive. The thickness of the LV wall increases from 300 μm to 400 μm, from HH27 to HH31, respectively.

The conotruncal banding model (CTB) results in an acute increase in ventricular pressure leading to an abnormal development. Miller et al. [30] showed that when the LV of Leghorn chicks with CTB is subjected to biaxial loading, the stress/strain values remain unchanged from HH27 to HH31. This contrasts with the healthy LV development, for which these values decrease as the embryo develops, supporting the hypothesis that mechanical uniaxial loading primarily regulates the trabecular aspect [30]. Another study on the CTB model reported ventricular dilation, thickening of the compact myocardium and trabeculae, and spiraling of trabecular course in the LV [65]. Since the ventricle is an anisotropic material, strains evaluated along different axes result in different values. For this review, we report the circumferential strain while briefly exploring longitudinal strains. The circumferential strain is evaluated for a circular vessel from the inner radius to the outer radius. Values compiled from literature are summarized in Table 2, along with their equivalent Carnegie stages, to allow for a timeline comparison across different species.

Tobita et al. observed the mechanical behavior of ventricle strains at LV and RV measured for stages HH21 to HH31 [10], along the circumferential and longitudinal directions. Tobita et al. observed that the strain consistently increased with embryonic stage. Epicardial wall strain at end of diastole at HH11 and HH27 for the circumferential and longitudinal orientations at LV and RV was also reported [68]. As the embryo develops, strain increases linearly along all axes [69]. Table 2 summarizes the strain levels reported in the literature from HH11 to HH35. It is observed that LV consistently has a lower longitudinal strain than RV and a higher circumferential strain than RV [10].

**Table 2:** Material properties of embryonic ventricles are summarized for the avian embryo. Regional properties of ventricles, epicardium, valve leaflets/cushions, atrioventricular region, myocardial wall, dorsal aorta, and atrium are compiled as available in the literature. Properties were evaluated at different embryonic stages primarily using the methods in Table 1. Reference sources (Ref.) are provided in the first column. LV: left ventricle, MPA: micropipette aspiration, RV: right ventricle, CTB: conotruncal banding, LAL: left-atrial ligation, FEM: finite element modeling, AV: atrioventricular, CS: Carnegie stage.

Ref.	Vascular Component	Parameter	Type	Stage		Value	Method
				HH	CS		
Ventricle Looping							
[43]	ventricle looping	Pressure (kPa)	systolic	16	12.6	0.133	Computational model and cuts
			diastolic			0.033	
		Stress (kPa)	Cauchy			2	
		strain	max			0	
			min			−0.2	
			bending			−0.2	
Epicardium							
[9]	epicardium	strain	max	16	12.6	0	epicardial beads
			min			−0.2	
			bending			−0.2	
		stress (kPa)	max			4	
		strain	max			−0.1	
			min			−0.2	
[68]	epicardial	strain	max circ	11	11	−0.1	triangular array
			max inner	12	11	0.1	
			systole bending	12	11	0.02	
			diastole bending	12	11	0	
Ventricle							
[67]	ventricle	strain	max	16	12.6	0.2	MPA
[30]	LV	thickness (microns)		27	17.5	300	uniaxial and biaxial testing
				29	19	400	
				31	20	425	
[63]	LV	thickness (microns)	compact layer	24	16	30	Micro-indentation and FEM
				29	19	40	
				34	21	45	
	RV			24	16	40	
				29	19	60	
				34	21	70	
[10]	LV	strain	circumferential	21	15	0.12	Beads
				27	17.5	0.23	
			end diastole	21	15	0.12	
	RV		circumferential	21	15	0.13	
	RV			27	17.5	0.23	
	LV			27	17.5	0.19	
[7]	ventricle	pressure (kPa)	systole max	21	15	0.2	Cuts, theoretical model, and Micro-pressure system
			diastole max	21	15	0.067	
[70]			max	24	16	0.06	
[65]	myocardial	circumferential stiffness constant	RV	27	17.5	4.3	Beads
			LV	27	17.5	7.8	
[19]	LV	pressure (kPa)	max diastole	29	19	0.631	FEM Servo-pressure
			max systole			0.062	
		stress (kPa)	von mises			1	Cuts
		strain	von mises			0.5	
[8]	ventricle	stress (kPa)	residual	12	11	27.2	
[71]	cardiac jelly	stiffness (N/m)	max	12	11	0.00225	
Valve Leaflet							
[49]	septal	strain energy density (Pa)	energy density	25	16.5	0.3	FEM
	mural			25	16.5	0.75	
	septal			29	19	0.85	
	mural			29	19	0.75	
	septal			34	21	1.5	
	mural			34	21	1	
Atrio Ventricular region							
[72]	AV region	modulus (kPa)	effective mod cushion	17	13	0.0001	Micro pipette aspiration
				21	15	0.001	
				25	16.5	0.004	
[41]	AV canal	stress (kPa)	Shear min	24	16	0.002	Immunofluorescence
			Shear min	28	18	0.002	
			Shear min	30	19	0.002	
Myocardial Wall							
[2]	myocardial wall	strain (%)	max	18	13.5	70	Doppler OCT
		strain rate (1/s)	rate			5	
		thickness (mm)				0.85	
Aorta Dorsal							
[4]	aortic (dorsal)	pressure (kPa)	range	27	17.5	0–0.180	LAL and Velocimeter pressure measurement
Atrium							
[41]	atrium	stress (kPa)	shear min	24	16	0.0128	Stress sensors and Immunofluorescence
				28	18	0.0118	
				30		0.0128	

Myocardial stiffness as an index of resistance to deformation, at stages HH21 and HH27, was measured by Tobita et al. [65] for LV and RV. The stiffness constant is a dimensionless quantity derived from the following equation: *σ* = *a* × *exp*(*bE*) and reported for the normal embryo at HH27. The stiffness constant of RV is 4.5 and 5.4 circumferentially and longitudinally, respectively. The corresponding values of stiffness constant for LV are 7.8 and 9.6, in which the longitudinal stiffness is higher than circumferential stiffness [65]. LV is stiffer that RV due to reduced volume load compared to LV.

At HH24, the end-diastolic stiffness reported by Stekelenburg et al. [20] was 0.6 mmHg/μL for a healthy embryo. A decrease in passive filling is observed for stiffer ventricular walls. The relative weight of the embryo remains constant between HH12 and HH29. The effective elastance obtained from pressure–volume loops indicates the contractility of the heart. Contractility, defined as the ability of a muscle tissue to shrink, is estimated to be 7.53 mmHg/μL [20]. HH21 has a higher diastolic stiffness than HH24, indicating a decrease in stiffness and increase in elasticity as the embryonic timeline progresses. As expected, stresses differ during the contraction and relaxation phases of the heart; at the end-diastole, for HH21, it was measured to be between 15–20 mmHg, whereas, at end-systole, it is between 100–150 mmHg [69].

Strains are measured for the ventricles at the outer curvature, the center, and the inner curvature for HH11 and HH12. While the microstructural properties are similar at the outer curvature and for the inner curvature, the circumferential direction has higher contractile stress levels. Strains at stages HH11 and HH12 are not significantly different but are higher in the central region for HH11 along both cardiac axes [68]. Strain values compiled from the literature show an increasing trend, as displayed in Figure 2.

Biaxial wall stress-strain relations also differ for the LV and RV as expected. Stress values peak at HH27 at any given strain level for both ventricles; however, for a CTB model, peak strain was observed at HH21 [65].

#### 3.1.2. Heart Valves

For atrioventricular valve leaflets at HH25 to HH34, the corresponding strain energy density functions were provided in [49], by utilizing two different experimental approaches. Deformable cantilevers were used for stages HH25 to HH34, whereas micropipette aspiration was used for stage HH36. It was observed that the strain energy density of the leaflet at HH36 is four times that at HH34. The strain energy density was also observed to increase by a factor of 2.5 from HH25 to HH29. Along the embryonic leaflet, the superior cushion region had a strain energy density of 0.44 Pa, while the inferior had a strain energy density of 0.34 Pa. Mural strain energies were observed to change from 0.6 Pa to 0.9 Pa between HH29 and HH34, respectively. Other available values from the literature are provided in Table 2.

#### 3.1.3. Aortic Arch and Vitelline Artery Properties

##### Geometry

Three paired aortic arch vessels appear in the development of early chick embryo. Wang et al. [73] studied the diameter of four of the six aortic arches and characterized the wall shear stress for these vessels. They observed that the midpoint diameters of the right arches are greater than the left and are in the range of 0.101 to 0.129 mm for HH18 and 0.113 to 0.138 mm for HH24, respectively [73]. Another study by Celik et al. evaluated the aortic arch diameters; at HH24, the maximum diameter was ~0.3 mm [74]. Lindsey et al. studied the diameter upon occlusion at HH18 and HH24 and found it to be ~0.2 mm [75].

##### Vascular Function, Pressure-Diameter Loops

**Figure 3:**
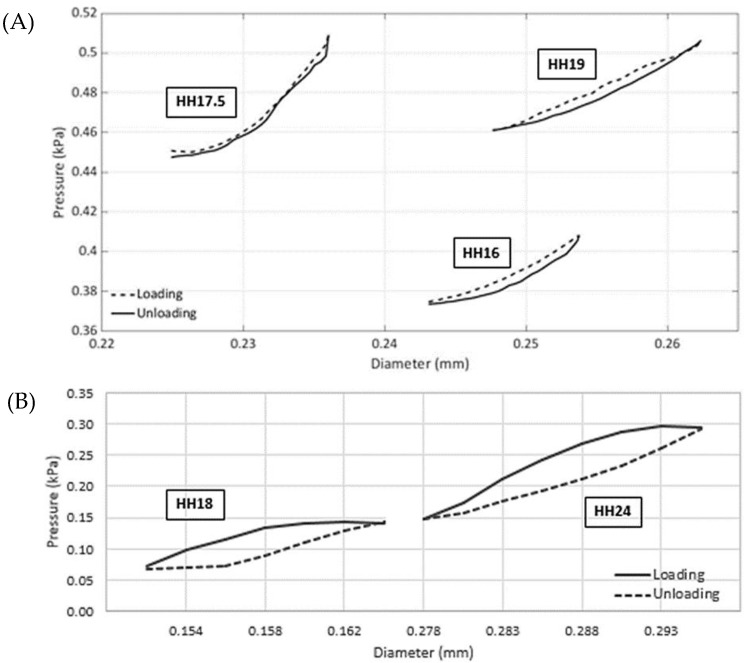
Representative pressure vs. lumen diameter loops of chick embryonic arteries acquired in vivo. (**A**). The vitelline arteries under loading and unloading conditions over the cardiac cycle. The data were collected for eight embryos (one representative sample shown) at three consequent stages of HH16, HH17.5, and HH19. (**B**). Right IVth aortic arch loading and unloading data over the cardiac cycle. The data were collected for five embryos (one sample shown) at two consequent stages of HH18 and HH24. Other sample data are available in the Supplementary Materials, and statistics are provided in Table 3. Data were acquired simultaneously using OCT and the servo-null pressure system described in Section 2.2.

In this section, employing the noninvasive approach described in Section 2.2 using pressure–diameter measurements acquired in vivo, a detailed mechanical analysis of vitelline arteries and the IVth aortic arch is presented. For both vessels, measurements indicate that the diameter increases nonuniformly with an increase in pressure (Figure 3). Another important finding is the existence of hysteresis during early stages as loading and unloading cycles are significantly different. Further studies are needed to quantify the level of hysteresis during later embryonic stages and how it is probably reduced in relation to the microstructure.

Pressure-diameter loops and the vessel thickness change during the cardiac cycle were used to obtain the Fung’s strain energy constants for both arteries using the method introduced by Donmazov et al. [12]. The values for the constants from Equation (2) were calculated and are presented in Table 3.

*Vitelline artery:* As presented in Table 3, the constant *c* is related to residual stress and increases with the stage of the embryo, as expected from pressure measurements and opening angle data. Constant *b*_1_ decreases as the embryo develops, and *b*_3_ nearly remains constant, while all other parameters (*b*_2_, *b*_4_, *b*_5_, and *b*_6_) increase during the growth of the vitelline artery. Due to in vivo measurements, a slight irregularity in loading curves is recorded. These parameters were evaluated under loading and unloading conditions. For example, it is observed that the constant *c* displayed a similar trend for both conditions, although the increment was steeper for loading. All other parameters had a similar trend for both loading and unloading conditions from HH16 to HH19.

Aortic arch vessels: Aortic arch vessels behaved differently as compared to vitelline artery during growth. The calculated Fung’s strain energy function parameters were similar for each stage, confirming the calculations noted in Table 3. The constant *c* increases with stage, *b*_3_ and *b*_4_ are nearly constant, and *b*_1_, *b*_2_, *b*_5_, and *b*_6_ decrease as the aortic arch grows. When these parameters were observed during loading and unloading conditions, parameter *c* followed the same trend for both with a steeper increase during loading. All other parameters had a similar behavior during both loading and unloading.

##### Effective Opening Angle and Residual Stress

Effective opening angle is derived from the loaded and unloaded ventricular morphology, as employed in the experiments conducted by Taber et al. on embryonic chick ventricles at HH stages 16, 18, 21, and 24 [69]. The effective opening angle increases as the stiffness of the vessel increases (31° at HH16), which is the residual stress component in one direction. Residual stress components along the orthogonal directions require additional incisions for the invasive approach. For the micron-size embryonic arteries, these experiments are very challenging to perform. As an alternative, herein, we present the results obtained from our non-invasive measurements based on Donmazov et al. [12]. Supplementary Figures S1 and S2 show the measured embryonic residual stress trends through the vessel wall along the radial (r), longitudinal (z), and circumferential (θ) directions. Residual stresses were computed for the vitelline artery and for the IVth aortic arch. It was found that, for the increased loading condition, residual stresses are greater than for unloading for both vitelline artery and aortic arch. In addition, an increase was observed in the values of residual stresses as the vessels grow.

**Figure 4:**
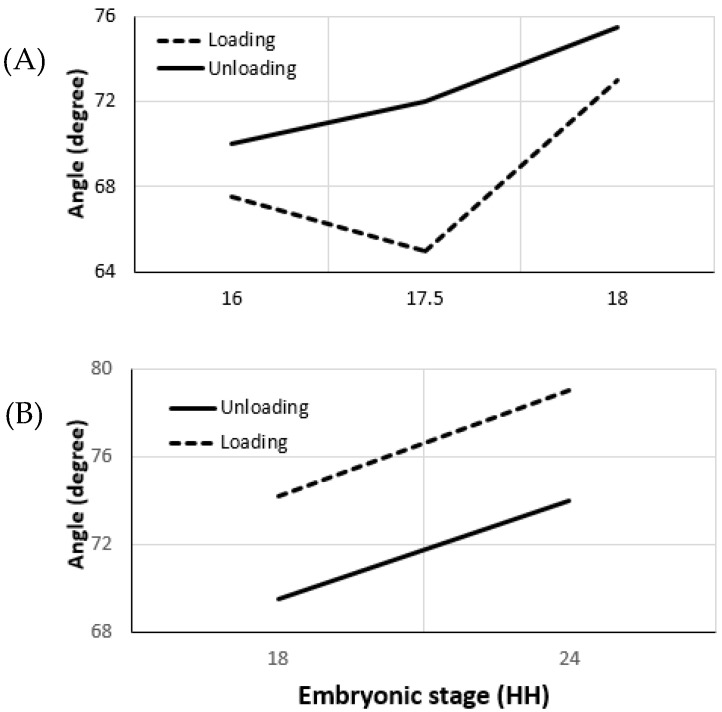
Changes in the effective opening angles of chick embryo arteries during early development are plotted. (**A**). Opening angle for vitelline arteries at stages HH16, HH17.5, and HH19 during loading and unloading conditions. (**B**). Opening angles for IVth right aortic arch at stages HH18 and HH24. Plots represent typical data collected over 13 chick embryos. Opening angles were calculated in our lab using the methodology presented in [12] using OCT and servo-null pressure data.

*Vitelline artery:* As plotted in Figure 4, it can be concluded that the vitelline artery at HH19 is stiffer than at HH16. Moreover, the sharp increase in its effective angle from stage HH17.5 to HH19 confirms this finding. Axial residual stress increases from 0.3 kPa to 0.4 kPa between the stages HH16 and HH19. The increase in residual stress in the circumferential direction and the radial direction is similar (~0.5 kPa).

*Aortic Arch vessels:* Vessel stiffness also increases for the IVth aortic arch from HH18 to HH24 (Figure 4). The effective angle is higher during the loading condition and agrees with the stiffness trend. Results indicate that the increase in axial residual stress for aortic arch vessels is much higher than the increase for the vitelline artery. The residual stress in axial direction at HH24 is almost twice the residual stress at HH18. While circumferential residual stress is 0.17 kPa at HH18, it almost doubles and becomes 0.33 kPa at stage 24. Although the increase in radial direction is not as high as in other directions, there is about a 0.1 kPa increase between the two stages studied [45].

Supplementary Figure S3 highlights that stress values are variable through the vessel wall and that the stress-strain relation is incremental. Both the vitelline artery and the aortic arch have the same characteristics in terms of residual stress distribution for loading and unloading conditions. Residual stress in the axial and radial directions increases from the inner vessel wall to the outer vessel wall, contrary to circumferential residual stress, which decreases.

##### Stress Distribution and Anisotropy

In the literature, ample information is provided for flow-induced wall shear stresses [76]; however, tissue stresses derived from Fung’s equation are unique to the present review as these values require a comprehensive structural soft-tissue analysis. As a general trend, stress increases with the growth of the vessel, and the slope of the stress-strain curves indicates slight stiffening. For both the vitelline artery and the aortic arch, the slopes of the stress-strain curves are higher for embryos at the later stages of development. As the embryo develops, it has increased circumferential rigidity compared to younger embryos. The von Mises stress distribution through the vessel wall is similar for relatively close stages. It is lowest at the inner wall of the vessel and highest at the outer wall for both vitelline and aortic arch vessels.

*Vitelline artery:* The von Mises stress at HH16 lies in the range of 0.17 to 0.22 kPa between embryos. For stages 17.5 and 19, the von Mises stresses are almost equal, in the range of 0.21 to 0.26 kPa. The von Mises stress is larger for loading than the unloading condition at all stages. Loading and unloading conditions have similar trends for von Mises stress values; however, HH19 has a higher von Mises stress than HH17.5.

*Aortic arch vessels:* At stage HH18, a large variation in stress values is recorded, ranging from 0.12 to 0.2 kPa and from 0.2 to 0.28 kPa for unloading and loading conditions, respectively. Stress values at HH24 are much higher than at HH18. The von Mises stress at HH24 lies in the range of 0.45 to 0.7 kPa.

### 3.2. Large Animal Models

For completeness, basic anatomical dimensions are first reported. In sheep, LV thickness at 80 days of gestation period is 2.2 mm, while, at day 127, it increases to 3.1 mm. When an artificial aortic banding was employed surgically, it was found that the thickness remains the same initially but increases significantly at later stages of embryonic development (7 mm at 140 days of gestation) [77]. The mitral valve diameter of the ovine fetus at 100 days of gestation is reported as 9.1 mm, while the aortic valve aortic annulus is 6.44 mm. An increase in the mechanical loading results in an increase in the mitral valve dimensions but a decrease in the aortic annulus [78].

Embryonic data on biomechanical properties of bovine and ovine cardiovascular components are limited. Despite the ample biomechanical data on mature porcine heart valves [28,79,80,81,82], limited embryonic studies have been conducted to our knowledge. Ovine arterial pressure at the fetal stage was measured for different gestational ages. While the mean aortic blood pressure in the first week of gestation is 2.5 mmHg [78], 5 weeks after gestation, it reaches 25 mmHg. At 116 days of gestation, the fetal descending aortic blood pressure is 41.1 mmHg [83,84] and the right-atrial pressure is ~2 mmHg, 115–121 days after gestation [83]. At the same gestational age (fifth week of gestation), the descending aortic blood pressure was found to be 64 mmHg [85]. The aortic pressure of the ewe was found to range between 39.2 and 41.0 mmHg, 126 days after gestation. Mean arterial pressure (MAP) remains nearly constant as the embryo develops; however, if there is an increased systolic load (40 mmHg for 8 days), the mean arterial pressure increases to 63 mmHg in 8 days [86]. A study conducted in 1989 measured the fetal blood pressure in ovine models to be ~44 mmHg [87], but no stage information was provided.

In [13], fetal and adult mitral valves were compared, where the maximum stress was observed to be 0.36 MPa for fetal porcine mitral valves (third trimester) and 1.48 MPa for 10 day old porcine mitral valves (Table 4). As the fetus develops, the thickness, the modulus of elasticity, and the stresses increase, as presented in Table 4, correlated with increased collagen content [13].

Cellular-level mechanical characteristics have been reported for large animals in the literature. Mononucleated and binucleated myocytes exhibit different lengths in ovine fetuses. At 135 days of gestation, mononucleated monocytes are 65 microns in LV and 69 microns in RV, with a width of 12 and 13 microns, respectively. Binucleated myocytes, on the other hand, have a length of 84 microns in LV and 92 microns in RV with a similar width to mononucleated myocytes [77]. Cardiomyocytes are generally unaffected by a reduction of load, but hyperplastic growth is affected by a reduced load in developing fetal hearts, as shown by a study conducted over a 100 day gestation period [78].

### 3.3. Human Embryonic Development

A reasonable estimate of human embryonic vascular properties can be obtained from umbilical vein and arteries. Maximum strain and stress values of the umbilical vein are 6 and 2.85 MPa, respectively [3]. The Young’s modulus and maximum stress were calculated to be 2.18 and 6.01 MPa, respectively. Since the umbilical tissue is anisotropic, an average global strain value is calculated over the complete region, which peaked at −14.4% for RV and −13.8% for LV [90]. As the fetus ages, the peak strain reduces. For instance, the LV strain increases from −13.8% in the first trimester to −10.8% in the second trimester. It is also observed that the LV strain is higher than the RV strain in the first trimester, but the RV strain is higher in the second trimester.

Throughout gestation, the global longitudinal strain decreases while the strain rates remain unchanged. The LV attains a higher strain value than RV with a mean value of 28% and 26%, respectively. The globally averaged longitudinal strain rate is 3.12 and 3.37 for RV and LV, respectively. A linear relationship can be developed between the gestational age and maximum strain [38].

Stress–strain characteristics measured in the radial direction for the umbilical vein show that, with the increase in artery wall thickness, the radial tensile strength increases until 750 μm, where the corresponding tensile strength is 7.5 N (Table 5) [42]. Similarly, the strength at 250 μm is 6 N, which implies that a lower thickness corresponds to lower stresses. Longitudinal tensile strength shows the same trend between 250 and 750 μm with values ranging from 9 to 21 N.

Another measure for estimating the material properties is by comparing the point of failure. In uniaxial or biaxial tensile testing, the material is stretched until failure which gives insight into the strength, elasticity, and stiffness of the material. Failure is caused either by direct and known stresses (primary failure) or by indirect, unknown stresses in the environment (secondary failure), both of which contribute to understanding the viscoelastic properties of the material. As an example, the umbilical cord consists of a component called the ringlet, as investigated by Rodriguez et al. [42]. Ringlet secondary failure occurs at a stress value of 0.5 MPa and remains constant as the thickness increases and the embryo develops However, the ringlet primary failure occurs at a higher stress value (2.1 MPa) for a lower umbilical arterial thickness and at a lower stress value (~0.4 MPa) as the thickness increases to 1000 μm. Similarly, longitudinal failure is highest at 250 μm at 3.4 MPa and lowest at 1000 μm. This shows that, as the embryo develops, it becomes stiffer and fails under lower stress values (Table 5).

The longitudinal tensile strength of the umbilical artery is the greater than the circumferential values (Table 5). The suture retention strength, as shown by Rodriguez et al., of the umbilical artery is highest (1.75 N at 1000 μm) and increases with increased thickness, as expected. Burst pressure evaluates the ability of a vessel to withstand high blood pressures. For the umbilical artery, the highest burst pressure is sustained at 1000 μm wall thickness, with a value of ~1500 mmHg [42]. Although the ability of the artery to withstand high pressure increases with thickness of the vessel, it decreases at a thickness of 750 microns to a value of 1250 mmHg. Umbilical cord arteries, however, were surprisingly found not to have any significant changes in the mechanical properties with an increase in gestational age [1]. Umbilical strain is a measure of deformability of the artery without any permanent deformation with respect to the original length. Although the strain increases with time, it is highest at the 30th week with a value of 1.52 [1].

### 3.4. Small Animal Models

For mice, the stages of embryonic development are defined in embryonic days (ED). Experiments conducted by Ishiwata et al. [88] for the end-diastolic area showed that the values increase as the embryo develops. At embryonic day 11.5 (ED11.5), the area of the LV and RV was found to be 0.77 mm^2^. The area increased from 0.8 mm^2^ at ED 12.5 to 1.29 mm^2^ at ED 14.5 [88].

Nonlinearity in the LV stiffness trend due to preconditioning behavior of resting myocardium is explained by strain softening [92]. Wistar-Hannover rats showed lower circumferential strain (−24.8%) than longitudinal strain (−19.3%). Pulmonary congestion resulted in higher longitudinal strain at about −10.1% and −13.8% circumferentially. Global strain rate showed a similar trend with circumferential and longitudinal strain rates of −4 s^−1^ and −4.7 s^−1^, respectively [93].

Alterations in myocardial stress and strain at septal walls reduce the functional capacity of the ventricle. These abnormal changes may indicate the presence and severity of CHD, as seen in Table 6. For example, myocardial strain levels for a heart with fibrotic infarction progressively increase to nearly twice the strain values of a healthy heart [14]. Table 6 reviews additional clinical cardiac abnormalities with significance in material properties.

Stresses were calculated for various cardiac regions, and it was seen that, when a fixed load is applied during uniaxial tensile testing, the circumferential, longitudinal, and radial stresses increase from 2.8 to 18.2 kPa, 1.5 to 9.7 kPa, and 0.1 kPa to 0.6 kPa, respectively. The increase in stress is 10-fold. Corresponding strain levels also increase from 0.100 to 0.138. RV deformations affect the LV diastolic mechanism and LV passive inflation. For both ventricles, stresses vary for the basal, equatorial, and apical and for the anterior, lateral, posterior, and septum regions. The highest stress is observed at the lateral equatorial region, reaching 3 kPa [98].

### 3.5. Mechanogenetic Regulation and Response

Vascular material properties are regulated through specific genetic pathways. In particular, the timescales involved in this tightly coupled system are not well established. In addition to the direct synthesis of matrix constituents, genes program material properties through biochemical means such as paracrine pathways to ensure that the cardiovascular system remains in its optimal condition. Cardiac looping and left–right asymmetry in ventricles are physiological features that are regulated by genes and play a role in the CHD and cardiac development [99]. Cyclic strain also plays a major role in cardiomyogenic differentiation of rat bone marrow stem cells as opposed to shear stress, and it increases cardiomyocyte-related markers [100].

The effect of shear stress has been studied in avian and mice embryos in the cardiovascular systems (umbilical arteries, and veins). It was observed that the gene expression profiles are altered by a shear stress-initiated release of prostacyclin and NO. Activated by changes in wall shear stress values [21,22,101,102,103,104,105,106], well-established mechanosensitive genes such as ET-1, NO, and eNOS are integral parts of the cardiovascular system. Growth hormone ET-1, for instance, is involved in vasoconstriction, and NO is involved in vasodilation [21], while KLF-2 is involved in vasculogenesis and angiogenesis [107]. Fluid shear stress is known to suppress endothelin 1 mechanosensitive genes [108]. ET-1 interacts with elastin and alters calcium content [109,110,111,112]. Elastin (Eln) in porcine embryos was evaluated, and its expression intensity was 42 in the third trimester. Despite the obvious differences in mitral and aortic valves following birth, these valves are identical in their genetic composition at the embryonic and fetal stages [113].

It is well established that arterial endothelial NO production and eNOS expression are controlled by shear stress [106]. KLF-2 expression is highest where the stress is highest. When the regulation of these genes is affected due to an inconsistency in the shear stress levels, the genetic risk factors for CHD increase, as in the NOS expression regulated by fluid shear stress [22,101]. Another study showed that the nature of force experienced influences the response of the endothelial genes [114]. Shear stress increases eNOS mRNA levels and cyclic stretch affects ET-1 mRNA levels. When applied simultaneously, the response of genes is significantly different from the individual effects.

Cardiac load also affects several genes and their expression levels. When the cardiac load is increased, a significant difference is observed in the levels of Notch-1, TGF-2, Wnt-2b, and BMP-1. For both mitral and aortic valves, these levels decline with the increase in load. Similarly, mRNA expression levels of elastin, type I collagen, and versican all decrease [115]. Cardiac malformations are known to depend on TGFβ levels [116] and shear stress affects KLF2 levels by activating the TGFβ/ALK5 signaling pathway [103,116,117]. In particular, miR-1 affects structural remodeling of the heart [118]. miR-128 regulates hyperplasia and Islet1 expression levels during cardiac regeneration [119]. Other genes such as Cadherin-11 and Fibirillin-1 affect thickness and arterial diameter. These genes also affect the calcification pathway in the cardiovascular system [120,121]. AGTR1, ACE, AGT, CYP11B2, and ADD1 are some factors that affect elasticity in the system [105]. MMP3, MMP9, and M235T affect the stiffness and impedance through the activation of factors. NFκB, MAP Kinase, MEK, and PI-3K affect the shear stress response in the system [31,122,123]. SMAD6 is indirectly associated with the thickness of the aorta despite not being mechanosensitive [124]. As such, abnormalities in SMAD levels can cause thoracic aortic aneurysms. The plasticity index is monitored by PKP2 in cardiac cells. Stress also activates IL33, which plays a role in end-stage cardiac failure [125,126]. RAAS is another important gene which is reported to influence the stiffness of the cardiac vessels [127]. Table 7 presents the mechanosensitive and mechanoresponsive genes that have been studied in literature.

## 4. Discussion

Among the several vascular components reviewed here, the myocardium has the highest stress concentrations at end-systole, while the residual stresses are responsible for further augmenting this stress level. At the early embryonic timepoint, the cardiac jelly present between the myocardium and the endocardium helps to enhance systole and diastole. Similarly, the stresses of the RV and LV are nonuniformly distributed at later stages [9]. As the embryo matures from HH16 to HH18, a 0.01 mm^2^ increase in the cross-sectional area of the tubular ventricle is observed. The LV cross-sectional area also increases from HH27 to HH31, together with the ventricular strain due to increased stress levels [67]. It was found that the LV is always thicker than the RV [63]. Similarly, ventricular length increases with development. The strain data show a similar trend for the ventricles [30]. Later stages experience lower stress levels in the ventricles between HH21 and HH27. It was also observed that circumferential strain is always higher than longitudinal strain [68]. RV has lower myocardial stiffness than the left in all cases and in all directions [65]. Compact layer thickness also increases as the chick embryo matures. This layer carries the highest stresses corelated with growth. Systolic pressure is significantly lower than diastolic pressure. Likewise, diastolic stiffness is also higher than systolic values. However, the diastolic stiffness reduces as the embryo develops [9,68,69].

Strain energy for developing septal heart valve leaflets increases as the embryo develops. The superior cushion of the heart valve leaflets experiences higher strain energy than the inferior. Myocardial mural strain energy also shows a similar increase with the embryonic development.

Longitudinal strength and suture retention strength for the umbilical arteries increase uniformly with the increase in thickness, while burst pressure increases nonuniformly, as shown by Rodrigue et al. [42]. It is also seen that, as thickness increases, primary and longitudinal failure occur at lower stress levels unlike secondary failure, which remains nearly constant.

While the mechanical properties of disease states were not the focus of this review, it is well established that the symptoms of fibrotic infarction include nearly doubled ventricular strain and increased myocardial stress [14]. Mechanical interventions and diseases can lead to similar effects. For example CTB shows increased pressure in the LV [19]; thus, we can conclude that conotruncal cardiac anomalies also cause increased pressure in the LV. As such, pulmonary congestion occurs when the longitudinal strain is higher than circumferential strain. Reduced strains in LV [16] and systolic strains [15], as well as increased stiffness [16], cause a hypertensive heart. Hypertrophy occurs when there is an increased ventricular wall thickness and stiffness [17].

Likewise, CHDs may be initiated when there are reduced strains in the ventricles; higher end-diastolic stresses and altered cross-fiber stresses can cause vascular aneurysms [18]. For example, verapamil suffusion occurs with decreased pressure in the ventricles with a compact and thinner myocardium [19]. In a recent study, the effects of various disease features on functionality of the heart were modeled [55]. These studies indicated that LV hypertrophy significantly increased LV pressure, strain, and stroke volume, leading to high mitral regurgitation valve velocities.

In this review, while the cellular-level bulk material properties were only briefly included, complex molecular structures and the material property changes during cellular differentiation deserve dedicated review efforts. For example, cardiomyocytes have a significantly lower value of strength and elastic modulus than hESCs (human embryonic stem cells), showing that the cardiomyocytes lose their strength and elasticity during their specialization, as summarized in Table 5.

## 5. Conclusions

Material properties of tissues, during development, growth, and disease states, affect how these tissues respond to mechanical forces and their microenvironment. In order to understand and control the vascular structure, the complexity of the cardiovascular system justifies an integrated approach that couples mechano-genetic characteristics with the material properties. Model organisms that possess a biventricular cardiovascular system broadly resemble the human circulation. Due to ethical reasons and experimental challenges, inferences from these model systems are informative for the development of the human heart. Therefore, the present study provides a look-up table of the embryonic material properties across species where human vascular data can be approximately referenced. As such, overall strain and stiffness values tend to increase with age of the developing embryo. It is also observed that the strain values for the LV are consistently higher than for the RV across species. Similarly, ventricular, pressures and size also continue to increase with age. While there is an increase in size, RV continues to grow faster than the LV, maintaining a larger ventricular size and thickness throughout the growth [88]. It is also important to note that the radial strength is generally considerably lower than the longitudinal strength in vascular vessels (Table 5). Genetic signaling also interacts with the mechanical characteristics through the paracrine pathways. Alternatively, studying the genetic patterns can explain changes in the material properties and the body’s reaction to these changes. The review also evaluated the consequences of malfunctioning genes and/or abnormal mechanical loading changes and their role in causing CHDs. It is hoped that these inferences concatenate the anatomical, genetic, and mechanical understanding of the heart and further inspire new interdisciplinary studies of complex cardiovascular system.

## Figures and Tables

**Table 1:** Summary of established mechanical techniques used to acquire the embryonic material properties and associated visualization methods.

Mechanical Methods and Numerical Models	Ref.	Visualization Methods	Ref.
Uniaxial/biaxial tensile testing	[30]	Optical coherence tomography	[31,32]
Invasive/noninvasive residual stress experiments	[33]	Epifluorescence/fluoroscopy	[34]
In vivo pressurization	[35]	Microscopy	[34,36]
Optical stretching and optical tweezers	[37]	Magnetic resonance imaging	[18]
Finite element modeling (FEM)	[3]	Echocardiograph	[38,39]
Cantilever based technologies	[29]	Confocal/two-photon microscopy	[19]
Strain energy and Gasser-Ogden-Holzapfel models	[40]	Scanning electron microscopy	[41,42]
Cuts	[43]	Histology	[44]
Micropipette aspiration with servo-null pressure measurements	[45]	Digital camera	
Beads	[9,10]	Radiology	
Micro-indentation, atomic force microscopy	[32,46]	Micro computed tomography	

**Table 3:** Estimated Fung’s strain energy function parameters (Equation (1)) for loading and unloading at HH16, HH17.5, and HH19 for the vitelline artery vessels and for the IVth aortic arch at HH18 and HH24 are tabulated. Sample numbers (*n*) are indicated for each artery type.

Vitelline Artery (*n* = 5)								Aortic Arch (*n* = 8)					
Loading				Unloading				Loading			Unloading		
HH	16	17.5	19		16	17.5	19		18	24		18	24
*c*	0.46 ± 0.03	0.51 ± 0.04	0.58 ± 0.04	*c*	0.43 ± 0.03	0.47 ± 0.04	0.57 ± 0.04	*c*	0.51 ± 0.03	0.61 ± 0.07	*c*	0.48 ± 0.02	0.57 ± 0.06
*b*1	4.68 ± 0.14	4.45 ± 0.14	4.23 ± 0.13	*b*1	4.49 ± 0.14	4.14 ± 0.13	4.1 ± 0.12	*b*1	6.51 ± 0.19	5.00 ± 0.25	*b*1	6.09 ± 0.18	4.68 ± 0.24
*b*2	2.81 ± 0.14	3.12 ± 0.15	3.28 ± 0.16	*b*2	2.69 ± 0.13	2.91 ± 0.14	3.18 ± 0.15	*b*2	2.53 ± 0.07	2.13 ± 0.07	*b*2	2.37 ± 0.07	1.99 ± 0.07
*b*3	0.65 ± 0.02	0.67 ± 0.02	0.65 ± 0.02	*b*3	0.63 ± 0.02	0.62 ± 0.02	0.63 ± 0.02	*b*3	0.63 ± 0.05	0.59 ± 0.06	*b*3	0.59 ± 0.05	0.55 ± 0.05
*b*4	0.37 ± 0.03	0.45 ± 0.04	0.51 ± 0.04	*b*4	0.36 ± 0.03	0.42 ± 0.03	0.50 ± 0.04	*b*4	0.32 ± 0.02	0.27 ± 0.03	*b*4	0.30 ± 0.02	0.26 ± 0.03
*b*5	6.04 ± 0.14	7.07 ± 0.17	7.86 ± 0.19	*b*5	5.80 ± 0.14	6.58 ± 0.16	7.62 ± 0.18	*b*5	6.40 ± 0.12	5.11 ± 0.12	*b*5	5.99 ± 0.11	4.77 ± 0.11
*b*6	1.47 ± 0.03	1.76 ± 0.04	1.86 ± 0.04	*b*6	1.41 ± 0.03	1.64 ± 0.04	1.81 ± 0.04	*b*6	0.98 ± 0.03	0.90 ± 0.03	*b*6	0.91 ± 0.03	0.84 ± 0.03

**Table 4:** Summary of the material properties for swine, rat, and *Xenopus* models during embryonic development. Embryonic stages for mice are recorded using the ED scale (embryonic day). ED0 is the day of fertilization, followed by ED1, ED2, etc. Similarly, for *Xenopus*, a scale measuring the number of hours post fertilization is used. As a general reference, CS is also provided. LV: left ventricle, RV: right ventricle, CS: Carnegie stage, ECG: electrocardiography, hpf: hours post fertilization.

Porcine						
Ref.	Organ	Parameter	Stage	CS	Value	Method
[13]	Mitral valve leaflet	thickness (mm)	Third trimester	-	0.4	uniaxial tensile testing
		stress (kPa)			7000	
		strain			0.35	
		ultimate stress (kPa)			0.400	
		modulus of elasticity (kPa)			200	
Rat						
Ref.	Organ	Parameter	Stage		Value	Method
			ED	CS		
[88]	Ventricle					Pulsed Doppler velocimetry and Micro pressure system
	Ventricle	Systolic pressure (mmHg)	10.5	7	3	
			11.5	8	5	
			12.5	9	6.5	
			13.5	10.5	8.2	
	LV	End diastolic area (mm^2^)	11.5	8	0.77	
	RV		11.5	8	0.77	
	LV		12.5	9	0.8	
	RV		14.5	11.5	1.29	
	Cardiac Myocyte					
	cardiac myocyte	Pressure (N/m)	2 days	-	0.75	
Xenopus						
[35]	myocardium	Stiffness (kPa)	48 hpf	-	10	FEM, cannulation, and pressurization
[89]	Heart tube	circumferential stress (kPa)	24–30 hpf	-	7	Computational

**Table 5:** Basic properties of human cardiovascular components at different gestational ages. Clinical measurement techniques and clinical image modalities are generally employed for data acquisition.

Ref.	Organ	Parameter	Type	Stage (Weeks)	Value	Method
Myocardium						
[90]	myocardium	strain (%)	RV global	1st trimester	14.4	Uniaxial tensile testing, FEM, and ECG
			LV global		13.8	
			RV regional		13.9	
			LV regional		13	
Ventricle						
[38]	LV	strain (%)	global	16–21	28.6	ECG
	LV			22–27	27.47	
	LV			28–38	26.61	
	RV			16–21	27.79	
	RV			22–27	26.48	
	RV			28–38	24.72	
	LV		systolic	21	−15	
	LV			28	−25	
	LV			34	−35	
umbilical vein						
[3]	umbilical vein	strain	derived from true stress and/or strain	-	4.1	FEM and uniaxial tensile testing
		elastic modulus (MPa)			4.5	
		stress (kPa)	max		6000	
		strain	max		0.9	
umbilical artery						
[1]	umbilical artery	stiffness (kPa)		25	57.89	uniaxial tensile testing and scanning electron microscope
				26–30	55.51	
				31–35	76.53	
				36–40	80.83	
		strain		25	1.33	
				26–30	1.52	
				31–35	1.39	
				36–40	1.41	
[42]		burst pressure (kPa)		-	200	
		strength (N)	suture retention	-	1.75	
		stress (kPa)		-	3500	
		strength (N)	longitudinal tensile	-	21	
		strength (N)	radial tensile	-	8	
Aorta						
[39]	aorta	Stiffness index	aortic compliance	25	0.7	Doppler flow profile and ECG
				30	0.5	
				35	0.25	
Valves						
[91]	aortic valve	Elastic Modulus (kPa)		21	4–5	Micro indentation
	Pulmonary valve				3–4	
human embryonic stem cells						
[37]	hESC	cardiomyocyte (kPa)	membrane stress	-	0.0013	optical stretching
			elastic modulus		0.0056	
			membrane stress		0.0005	
			elastic modulus		0.014	

**Table 6:** General material property trends for selected cardiac malformations. CTB: conotruncal banding, LV: left ventricle, RV: right ventricle, CHD: congenital heart disease.

Fetal Malformation	Change in Material Property	Ref.
Fibrotic infarction	Doubled ventricular strain and increased myocardial stress.	[14]
Conotruncal defects	Increased ventricular pressure	[19]
pulmonary congestion	Higher longitudinal strain compared to the circumferential direction	[94]
Hypertensive heart	Increased stiffness	[15]
	Reduced strains in LV	[16]
	Reduced systolic strains	
Hypertrophy	Increased ventricular wall thickness and stiffness	[17]
aneurysm	Higher end diastolic stresses and cross-fiber stresses	[18]
Marfan syndrome	Enlargement and weakening of heart muscles	[95]
Loeys-Dietz syndrome	Enlargement of aorta	[96]
Ehlers-Danlos syndrome	Reduced elasticity, strength, and stiffness of the aortic vessels	[97]
Fibrotic infarction	Decreased ventricular pressure with compact and thinner myocardium	[19]

**Table 7:** Mechanical property phenotypes and the response of mechanosensitive genes that play a critical role in structural cardiovascular development are summarized. Mechanosensitive genes are activated as a response to mechanical changes, while mechanoresponsive genes cause a mechanical change in the tissues. The effect of abnormal gene/pathway signaling is associated with cardiovascular system defects. WT: wild type, KO: knockout. KI: knock-in, M: mutant, R: review.

Gene	Organ	Defect	Mechanosensitive	Indirect Alterations	Mechanical Properties Altered	Ref.	GP
Fibulin 4 coded by EFEMP2 gene	Large conduit arterial walls in mice	Ascending Aortic aneurysms, loose skin, bent forelimb, tortuous artery, and pulmonary emphysema	Interacts with elastin directly	-	Alters elastin, binds to calcium	[109]	KI
						[110]	KO
						[111]	KI
						[112]	KO
Endothelin 1 (ET1)	Human umbilical vein endothelial cells		Reacts directly to shear stress and cyclic stretch		Shear stress and cyclic stretch	[114]	WT
Elastin coded by Eln	Mouse aortic walls	Arterial stenosis, hypertension	Direct interaction	-	Alters elastic fibers, thickening and arterial tortuosity	[110]	KO
						[128]	M
						[129]	KO
miR-1	Cardiac contractile function in mice	Damage in sarcomere assembly	-	Targets UTRs of MYLK3, CALM1, and CALM2	Affects structural remodeling of the heart	[118]	KO
VEGF	Endothelial cells	Matrix stiffens	-	In turn effects MMP activity	Stiffness, intima	[104,130]	M
Cadherin-11	ECM in aorta in mice	Cardiac dysfunction in valves	-	Reduced Sox9 activity, β1 integrin expression, and RhoA-GTP	Increases thickness and alters stress fibers, causes calcification	[120]	KO
						[121]	KI
Fibirillin-1	Mice arteries	Mutation causes Narrowing			Affects arterial diameter	[128]	M
NOS-3, KLF-2, ET-1 (can be altered by changing trichloroethylene doses) [131]	Chick, bovine, mice embryonic cardiovascular system		Shear stress induced	KLF2 indirectly activated by TGFβ	Activated by shear stress	[104]	M
						[101,106]	WT
TGFβ	Embryonic endothelial cells (human)	Cardiac malformations	Activated by shear activities	Can be affected by fibulin deficiency	Directly activated by shear stress	[103,116,117]	WT, M
ROBO4	Bicuspid aortic valve and thoracic aortic	CHD and aneurysm				[132]	KO
Notch1	Mice aorta	Ascending Aortic Aneurysm				[133]	KO
AGTR1, ACE, AGT, CYP11B2, ADD1	Human artery, vascular	CHD		Indirect association	Elasticity	[105]	R
MMP3, MMP9, M235T	Human artery, mice vascular	CHD		Indirect association	Stiffness and impedance	[105,134]	R
NFκB	Vascular response in mice		Direct		Activated by shear stress	[123]	R
MAP Kinase	Blood vessels		Shear activated or stretch activated		Shear stress and stretch	[8]	
MEK, PI-3K	Ovine fetoplacental artery endothelial cells			Activated by eNOS (indirectly activated by stress)	Shear stress	[31]	WT
SMAD6	Thoracic aorta and bicuspid valve in humans	Thoracic aortic aneurysm		Indirect	Thickness	[124]	M
PKP2	Cardiac cells inMice			Indirect, affects miR200b first	Knockdown causes reduced stress and work of detachment, increases plasticity index	[135]	KO
IL33	Myocardium in mice and humans	Failing heart	Induced by mechanical stress		Stress	[126]	KO
						[125]	WT
miR-128	Cardiac ECM	Hyperplasia		Regulates hyperplasia and Islet1		[119]	KO
RAAS	Cardiac vessels	Vascular hypertrophy		Regulates stiffness	Stiffness	[127]	R

## Data Availability

All data acquired that support the findings of this study are available on request from the corresponding author.

## Supplementary Materials

The following supporting information can be downloaded at: https://www.mdpi.com/article/10.3390/jcdd9020064/s1, Figure S1: Computed Cauchy residual stress values for the vitelline vessels in chick embryo at HH16, HH17.5 and HH19 during physiologic loading and unloading conditions. Representative of one of the 8 embryos. calculated using the methodology presented in [12]; Figure S2: Computed Cauchy residual stress values for the chick embryo fourth aortic arch at HH18 and HH24 during physiologic loading and unloading conditions. Representative of one of the 5 embryos. calculated using the methodology presented in [12]; Figure S3: Stress strain relation for TOP: the vitelline vessels in chick embryo at HH16, HH17.5 and HH19 during physiologic loading and unloading conditions. Representative of one of the 8 embryos. BOTTOM: fourth aortic arch in chick embryo at HH18 and HH24 during physiologic loading and unloading conditions. Representative of one of the 5 embryos.
