# Supplementary material for: Soft-Tissue Material Properties and Mechanogenetics during Cardiovascular Development
Source: J Cardiovasc Dev Dis. 2022 Feb 21;9(2):64. doi: 10.3390/jcdd9020064 (PMC8876703; doi:10.3390/jcdd9020064)

SUPPLEMENTARY FIGURES

Figure S1: Computed Cauchy residual stress values for the vitelline vessels in chick embryo at HH16, HH17.5 and HH19 during physiologic loading and unloading conditions. Representative of one of the 8 embryos. calculated using the methodology presented in Ref. [1].

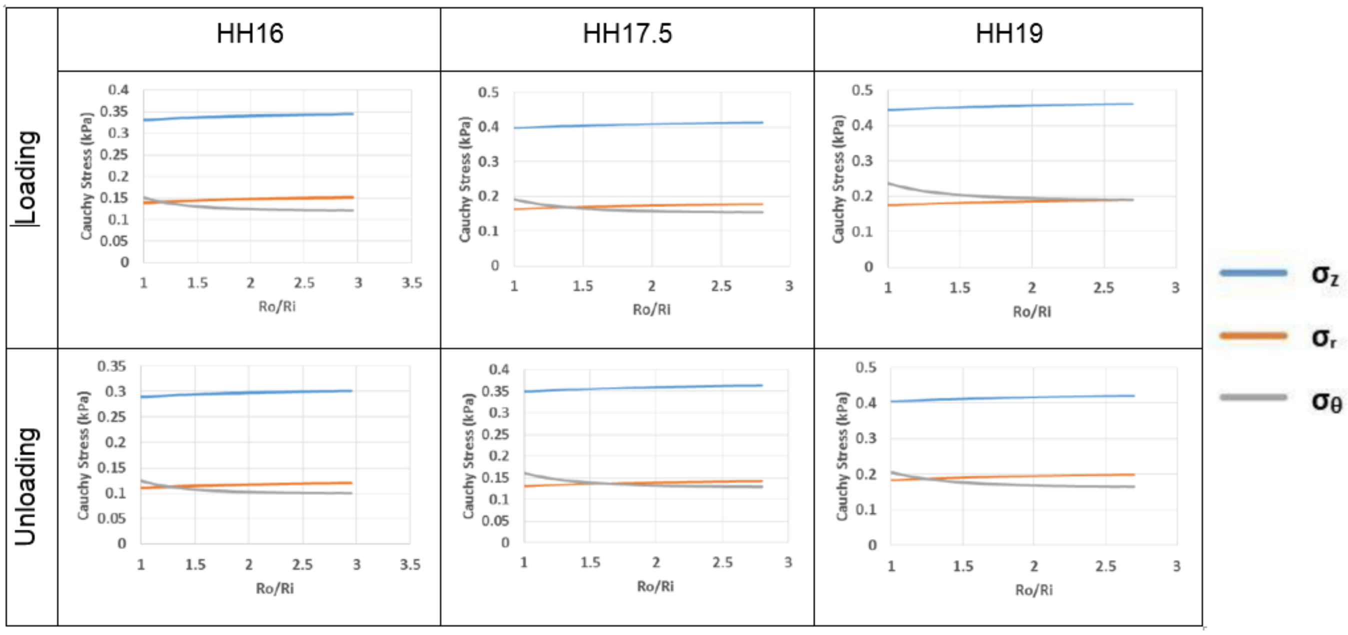

Figure S2: Computed Cauchy residual stress values for the chick embryo fourth aortic arch at HH18 and HH24 during physiologic loading and unloading conditions. Representative of one of the 5 embryos. calculated using the methodology presented in Ref [1].

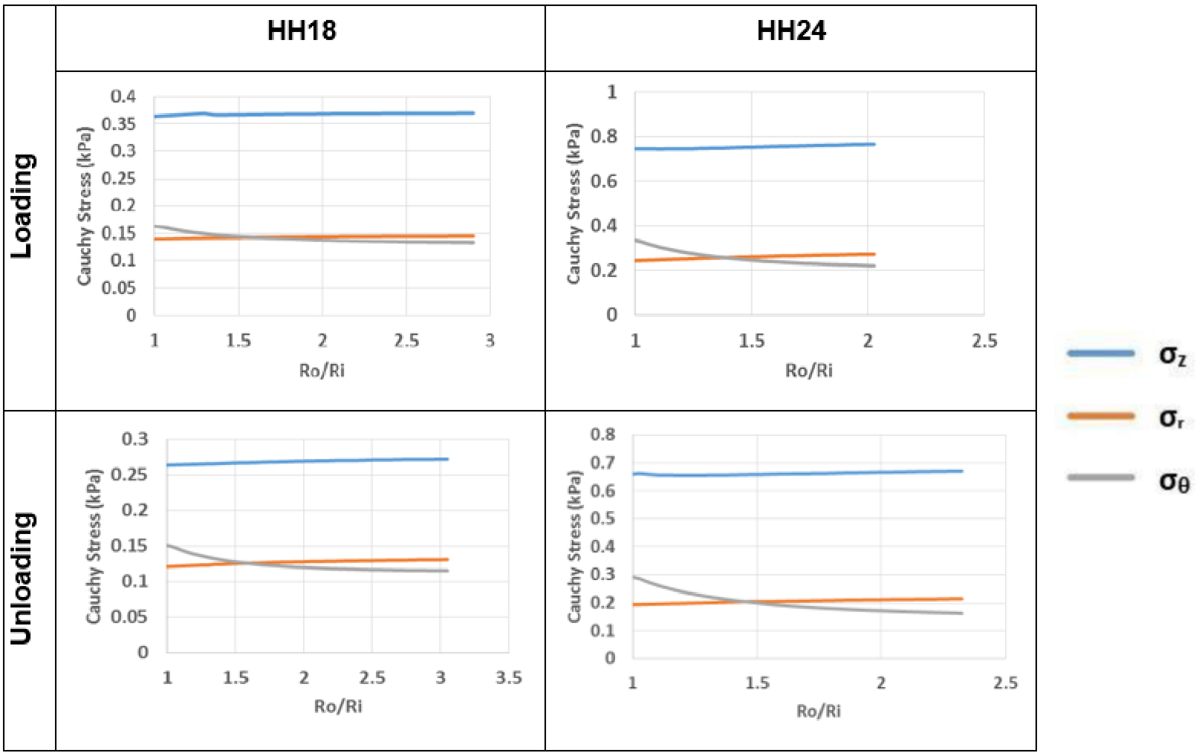

Figure S3: Stress strain relation for TOP: the vitelline vessels in chick embryo at HH16, HH17.5 and HH19 during physiologic loading and unloading conditions. Representative of one of the 8 embryos. BOTTOM: fourth aortic arch in chick embryo at HH18 and HH24 during physiologic loading and unloading conditions. Representative of one of the 5 embryos.

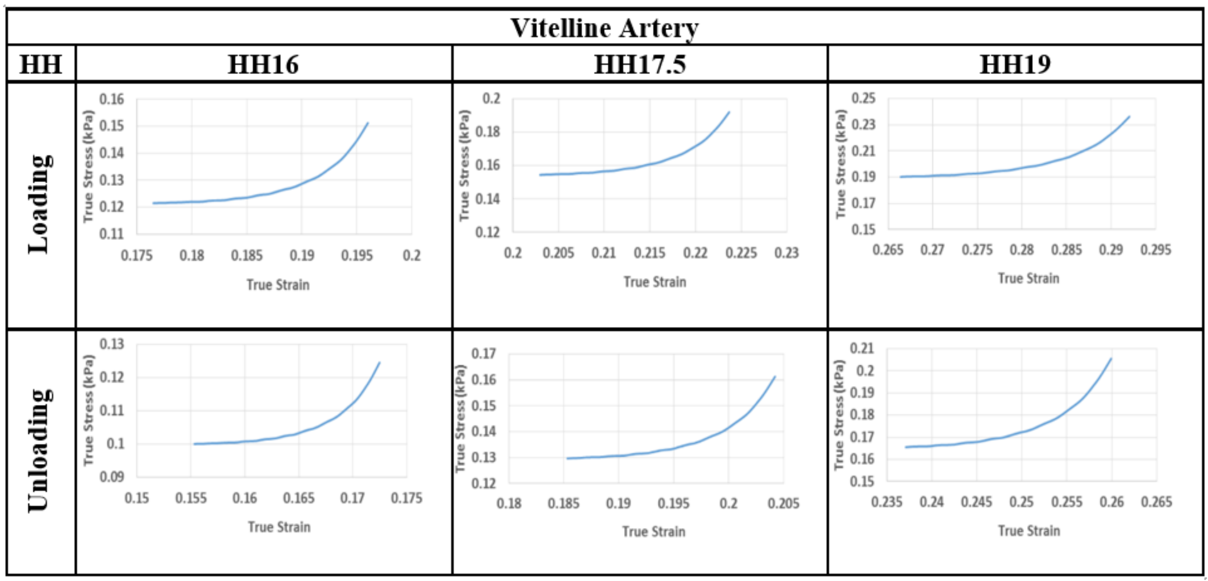

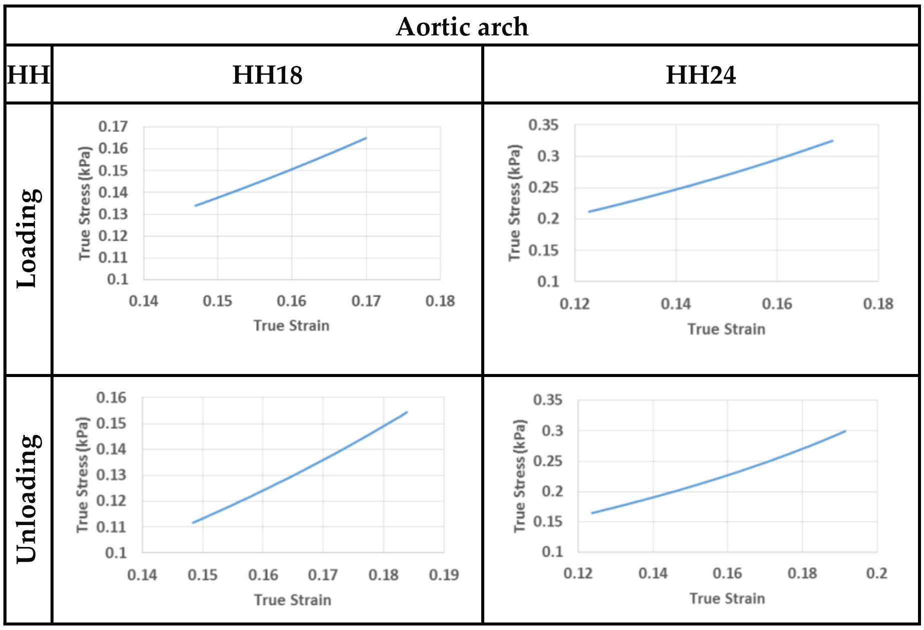

Supplement: Supplementary file 1 [file jcdd-09-00064-s001.zip › jcdd-1471211-supplementary.pdf]
